# Effects of Beta-Blockers on Heart Failure with Preserved Ejection Fraction: A Meta-Analysis

**DOI:** 10.1371/journal.pone.0090555

**Published:** 2014-03-05

**Authors:** Feng Liu, Yanmei Chen, Xuguang Feng, Zhonghua Teng, Ye Yuan, Jianping Bin

**Affiliations:** Department of Cardiology and National Key Lab for Organ Failure Research, Nanfang Hospital, Southern Medical University, Guangzhou, China; Tokai University, Japan

## Abstract

**Background:**

Effects of beta-blockers on the prognosis of the heart failure patients with preserved ejection fraction (HFpEF) remain controversial. The aim of this meta-analysis was to determine the impact of beta-blockers on mortality and hospitalization in the patients with HFpEF.

**Methods:**

A search of MEDLINE, EMBASE, and the Cochrane Library databases from 2005 to June 2013 was conducted. Clinical studies reporting outcomes of mortality and/or hospitalization for patients with HFpEF (EF ≥ 40%), being assigned to beta-blockers treatment and non-beta-blockers control group were included.

**Results:**

A total of 12 clinical studies (2 randomized controlled trials and 10 observational studies) involving 21,206 HFpEF patients were included for this meta-analysis. The pooled analysis demonstrated that beta-blocker exposure was associated with a 9% reduction in relative risk for all-cause mortality in patients with HFpEF (95% CI: 0.87 – 0.95; P < 0.001). Whereas, the all-cause hospitalization, HF hospitalization and composite outcomes (mortality and hospitalization) were not affected by this treatment (P = 0.26, P = 0.97, and P = 0.88 respectively).

**Conclusions:**

The beta-blockers treatment for the patients with HFpEF was associated with a lower risk of all-cause mortality, but not with a lower risk of hospitalization. These finding were mainly obtained from observational studies, and further investigations are needed to make an assertion.

## Introduction

Epidemiological data reveals that approximately 50% of chronic heart failure (HF) patients have normal or only mildly impaired left ventricular ejection fraction (EF), which is referred to as the HF with preserved EF (HFpEF) patients [1]. As the life expectancy increases and the population ages, the prevalence of HFpEF continues to increase [2]. More importantly, the prognosis of HFpEF patients remains poor, which is similar to that of HF patients with reduced ejection fraction (HFrEF) [3]. Thus, HFpEF is a growing major problem in public health in the world. However, efficacious therapies on HFpEF have not been well established.

Beta-blockers are a kind of drugs that inhibit sympathetic nervous system activity. It has been shown that patients with HFrEF receive significant benefits from the treatment [4], [5]. In contrast, the benefits of beta-blockers on mortality and hospitalization in patients with HFpEF have not been confirmed [6]. Currently, there is no consensus on the effect of beta-blockers in HFpEF. For instances, some observational studies demonstrated the beta-blockers treatment decreased the risks of all-cause mortality in the HFpEF patients [7]–[9], while the reduction was not observed in the sub-analysis of SENIORS trial [10] and J-DHF trial [11]. However, a small observational study has suggested that the prescription of beta-blockers increases the risk for hospitalization in the HFpEF patients [12]. Although a previous meta-analysis addressed the effects of pharmacotherapies (including beta-blockers) in the HFpEF patients [13], they used the threshold of an EF ≥ 35% as the diagnosis of the HFpEF patients, which is usually not considered “preserved.” In addition, several studies have been published since the previous meta-analysis was performed.

Given the limited evidence and uncertain effects of beta-blockers in the patients with HFpEF, this meta-analysis summarized the current data from randomized controlled trials (RCTs) and observational studies (OSs) to determine the impact of the beta-blockers treatment on mortality and hospitalization in the patients with HFpEF (an EF ≥ 40%).

## Methods

This meta-analysis was performed and reported according to the Preferred Reporting Items for Systematic Reviews and Meta-Analyses (PRISMA) [14] and the reporting Meta-Analyses of Observational Studies in Epidemiology (MOOSE) [15].

### Literature search

We conducted MEDLINE, EMBASE, and the Cochrane Library databases searches for the published clinical studies from 2005 through June 2013 using the following search terms: 1) heart failure with preserved ejection fraction or heart failure with normal ejection fraction or diastolic heart failure, 2) beta-blockers. Our literature search was limited to studies involving human subjects and those published in English. We manually searched the references that were cited in other relevant publications.

### Inclusion criteria

Inclusion criteria were: (1) assessment of the effectiveness of beta-blockers in the patients with HFpEF which had an EF ≥ 40%, (2) randomized controlled trials or nonrandomized controlled studies that provide information on the mortality and/or hospitalization, (3) studies that had a non-beta-blockers control group, (4) the duration of follow-up was at least 6 months.

### Data extraction

Information about the study and patient characteristics, methodological quality, intervention strategies, and clinical outcomes was systematically extracted separately by two reviewers. Disagreements were resolved by consensus.

### Methodological Quality

The quality of random control trial included was assessed by the Jadad quality scale [16]. The quality of the observational studies was evaluated by Newcastle-Ottawa Scale tool (available at: http://www.ohri.ca/programs/clinical_epidemiology/oxford.asp).

### Statistical Analysis

The relative risks (RRs) and 95% CI were used as the common measure across the studies. The hazard ratios (HRs) were considered equivalent to RRs [17]–[19]. If the effect estimates were not available in the studies included, the RRs were calculated by using the following formula: RR  =  Probability of events given treatment/Probability of events given no-treatment. If the studies provided the adjusted estimations, they were directly used in the meta-analysis. Statistical heterogeneity was tested by the Cochran Q statistic and reported as I^2^-value for every outcome [20]. The RRs were pooled using the fixed-effect models (Mantel-Haenszel method) in the absence of heterogeneity among studies (I^2^-value less than 50%). In the presence of heterogeneity, the RRs were pooled using a random-effects model (DerSimonian and Laird method) [21]. The publication bias was assessed by Begg’s test [22]. A significance level of alpha  =  0.05 was used.

A sensitivity analysis was conducted by removing one study from the total studies in each round and evaluating the influence of each single study on the primary meta-analysis result. The outcomes of all-cause mortality according to the selected study characteristics were assessed by subgroup analysis. All analyses were performed by statistical program Stata (version 11.2, Stata Corp, College Station, Texas) and R software (version. 3.0.1, available at: http://www.r-project.org/).

## Results

### Search results

**Figure 1:**
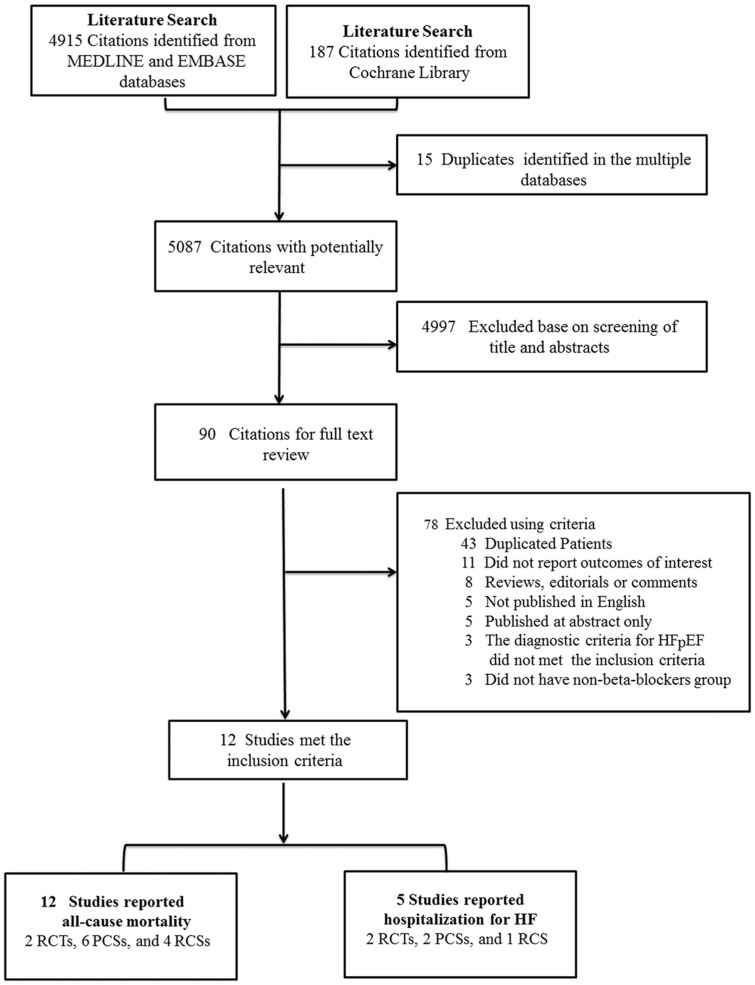
Flowchart of study search and selection in this meta-analysis. PCSs, prospective cohort studies; RCSs, retrospective cohort studies; RCTs, randomized controlled trials;

Figure 1 displays the flow diagram of study selection. Our initial search yielded 4,915 citations from MEDLINE and EMBASE databases, and 187 citations from Cochrane Library. After screening the titles and abstracts of all studies identified by the search strategy, 90 potentially relevant articles were further screened for the eligibility. Finally, 2 randomized control trials [10], [11] and 10 observational studies [7]–[9], [12], [23]–[28] were included.

### Characteristics and quality of study included

The characteristics of the studies included are presented in Table 1. Of the 12 studies included, 2 studies were randomize-controlled design [10], [11] (one from the sub-analysis), 6 studies were prospective cohort design [7], [12], [23]–[26], and 4 studies were retrospective design [8], [9], [27], [28]. The definition of HFpEF differed across the included studies, with an EF ≥ 40% in 5 studies and EF ≥ 50% in 7 studies. 4 studies included the elderly HFpEF patients only.

**Table 1:** Studies characteristics of the included studies.

Study	Year	Study design	Definition of HFPEF (LVEF)	Entry age (years)	Sample size	Beta-blockers group (N)	Non-beta-blocker group (N)	Mean follow-up (years)	Outcomes	estimate effect	Adjusted
**SENIORS ** [**10**]	2009	RCT	> 40%	>70	643	320	323	1.75	All-cause mortality and HF hospitalization	HR	Yes
**J-DHF** [**11**]	2013	RCT	> 40%	> 20	245	120	125	3.2	Mortality and hospitalization for HF	RR	Yes
**Fukuta H** [**23**]	2005	PCS	≥ 50%	NA	137	68	69	1.75	Death	RR	Yes
**Chan, J. D** [**24**]	2005	PCS	≥ 40%	> 65	342	NA	NA	2.3	All-cause mortality	HR	Yes
**Grigorian SL ** [**25**]	2005	PCS	> 50%	NA	416	98	318	4.57	Death	HR	Yes
**OPTIMIZE-HF** [**26**]	2009	PCS	> 40%	NA	4153	1621	2532	1	Mortality or readmission	HR	Yes
**Farasat SM ** [**12**]	2009	PCS	≥ 50%	> 18	66	43	23	0.5	All-cause mortality and HF hospitalization	RR	No
**Dobre D** [**7**]	2010	PCS	≥ 40%	> 30	443	227	216	2.08	All-cause death	HR	Yes
**Tehrani F** [**27**]	2008	RCS	≥ 50%	> 80	142	51	91	5	Death	RR	No
**R Shah ** [**8**]	2008	RCS	> 50%	> 65	13533	4562	8971	3	Death	RR	Yes
**NevzorovRV ** [**28**]	2012	RCS	≥ 50%	> 18	345	154	191	2	Mortality	HR	Yes
**El-Refai M** [**9**]	2013	RCS	≥ 50%	> 18	741	570	171	2.1	Death or hospitalization	HR	Yes

HR: Hazard ratio; PCS: prospective cohort study; RCS: retrospective cohort study; RCT: randomized controlled trial; RR: relative risk; NA: not available.

Among a total of 21,206 patients, 7,834 patients were in the beta-blockers group and 13,030 patients in the control group (Note: Chan’s study did not provide the exact data of the two groups). There were only 5 studies that provided the data regarding hospitalization [9]–[12], [26]. The mean follow-up period, ranging from 0.5 to 4.57 years, was similar between the beta-blockers and the non-beta-blockers group. The effect estimations of hazard ratios (HRs) were provided in 7 studies and RRs in 5 studies, which were adjusted for the baseline characteristics.

Of the two RCTs included, the Jadad score was 4 in the SENIORS trials and 2 in the J-DHF trials. The quality of the included OSs assessed by Newcastle-Ottawa Scale tool was displayed in Table S1 (median score, 7; range, 5 to 8).

### Patient characteristics

Patient characteristics of the studies included are presented in Table 2. The mean age of the patients with HFpEF was 77.7 years. Among them, 59.8% were female with the mean LVEF of 55.8%. Ischemic etiology was the primary cause for HF in 48.3% of the patients. The prevalence of hypertension, diabetes mellitus, atrial fibrillation and chronic obstructive pulmonary disease (COPD) was 70.7, 37.0%, 35.5% and 37.8%, respectively. Baseline medication included ACEI/ARB in 50.3% of the patients, diuretics in 75.6%, nitrates in 37.3%, and digoxin in 22.3%.

**Table 2:** Patients characteristics of the included studies.

Study	SENIORS[10]†	J-DHF[11]	FukutaH[23]	Chan, J. D[24]‡	Grigoria SL[25]	OPTIMIZE-HF[26]	FarasatSM[12]	Dobre D[7]	Tehrani F[27]	R Shah[8]	Nevzorov R[28]	El-Refai M[9]
**Year**	2009	2013	2005	2005	2006	2009	2009	2007	2008	2008	2012	2013
**Mean age, years** *	76(5)	72(11)	65(14)	80(5)	73(10)	81	71(13)	78(10)	87(5)	80	77(11)	71(12)
**Type of beta-blockers**	Nebivolol	Carvedilol	NA	NA	NA	NA	NA	NA	NA	NA	NA	NA
**Dose of beta-blockers**	Starting with 1.25mg/d to 10mg/d	7.5 mg/d	NA	NA	NA	NA	NA	NA	NA	NA	NA	NA
**Female,%**	49.9	42	57	50.3	51	31.6	8.2	55.5	69	70	58.8	60.6
**LVEF,%**	49(10)	63(11)	62(7)	NA	>50	NA	NA	≥40	60(10)	NA	NA	57 (5)
**History of MI,%**	34.4	NA	NA	60.5	NA	NA	NA	36.1	44	21	13.3	NA
**Ischemic etiology, %**	76.9	14.7	58	84.7	41.1	NA	48.5	NA	44	48	51.3	25.5
**Hypertension,%**	77.7	80.4	80	63.7	60.8	NA	89.4	49.4	75	71	69.8	73.4
**Diabetes mellitus,%**	24.3	3.6	23	16.8	26.9	36.6	54.5	28.7	26	39	40	46.4
**Atrial fibrillation (%)**	36.6	40.8	NA	23.6	NA	35.2	NA	44.5	NA	NA	44.9	29.8
**COPD (%)**	NA	NA	NA	14.7	NA	28.2	NA	28.4	NA	33	NA	39
**Anemia (%)**	NA	NA	NA	NA	37.8	19.7	NA	54.4	54	NA	32.5	NA
**Medication**												
**Statin**	13.3	NA	NA	12.7	NA	28	NA	NA	22	17	23.7	NA
**ACEI/ ARB, %**	91.5	23.3	55	44.5	50.5	58.2	NA	77.4	47	47	46.96	26
**Aspirin,%**	NA	20.4	NA	65.8	28.1	48.9	NA	NA	NA	41	36.8	NA
**Diuretics,%**	83.1	60	50	52.9	66.1	80.4	NA	88	62	NA	48.13	NA
**CCB,%**	NA	NA	27	27.4	31	NA	NA	15.3	33	NA	25.2	NA
**Nitrates,%**	NA	NA	NA	45.9	36.8	NA	NA	46.5	20	NA	24.7	NA
**Digoxin,%**	NA	20.4	NA	36.3	22.1	21.9	NA	22.1	30	NA	11.6	NA
**Mineralocorticoid receptor blockers,%**	5.6	23.3	NA	NA	NA	8.0	6.1	40.6	NA	NA	20.0	NA

*The values were given as mean (standard deviation), †the values were presented as the patients with an EF ≥ 35% among the participants who used the beta-blockers treatment, ‡the values were presented as the participants who used the beta-blockers treatment. ACEI: angiotensin-converting enzyme inhibitor; ARBs: angiotensin-receptor blockers; CCB: calcium channel blockers; COPD: chronic obstructive pulmonary disease; LVEF: left ventricular ejection fraction; MI: myocardial infarction; NA: not available.

### Effect of beta-blockers on all-cause mortality

**Figure 2:**
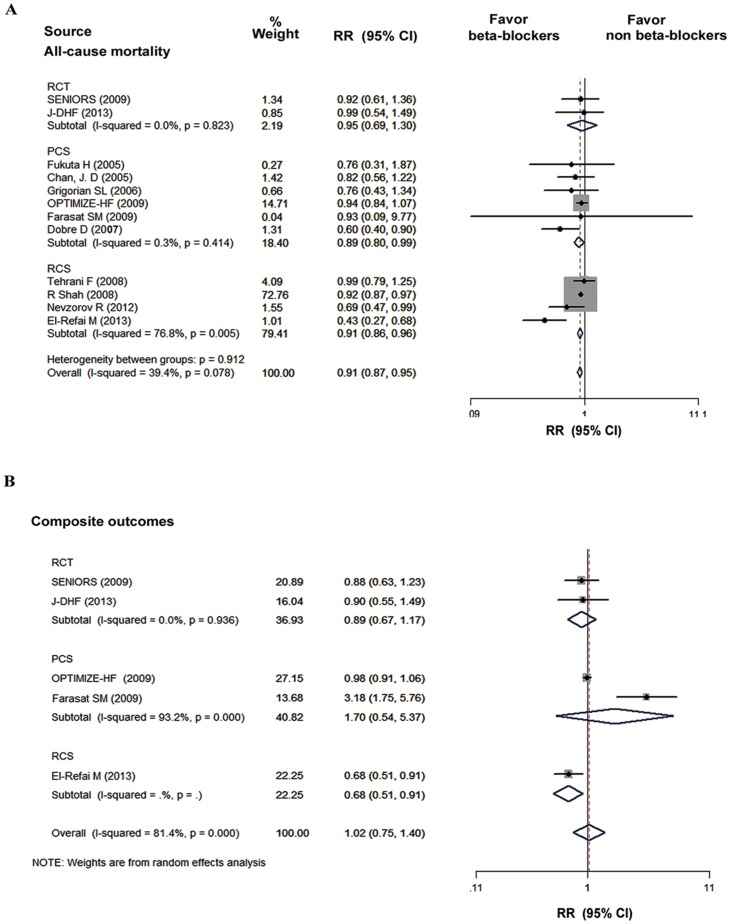
The pooled analyses of all-cause mortality and composites outcomes in beta-blockers group versus non-beta-blockers group. CI, confidence interval; PCS, prospective cohort study; RCS, retrospective cohort study; RCTs, randomized controlled trials; RR, relative risk.

Figure 2 displays the results of the pooled analyses of all studies that reported the outcomes of all-cause mortality and composite outcomes. Of the 12 studies that reported the mortality and hospitalization, only 4 studies showed that beta-blocker treatment was associated with a significantly lower risk of all-cause mortality in HFpEF patients. There was a 5% decrease in risk of all-cause mortality from the RCT (I^2^ = 0%, RR, 0.95; 95% CI, 0.69 to 1.30; P = 0.73), a 11% reduction from the prospective cohort studies (I^2^ = 0.3%, RR, 0.89; 95% CI, 0.80 to 0.99; P < 0.05), and a 22% reduction from the data of retrospective cohort studies (I^2^ = 76.8%, RR, 0.91; 95% CI, 0.86 to 0.96; P < 0.05). Overall, the treatment with beta-blockers was associated with a significant reduction in the risk for the mortality compared with the non-beta-blockers group (I^2^ = 39.4%, RR, 0.91; 95% CI, 0.87 to 0.95; P < 0.05). However, the composite outcomes of mortality and hospitalization was not affected by the beta-blockers treatment (I^2^ = 81.4%, RR, 1.02; 95% CI, 0.75 to 1.40; P = 0.88). No evidence for publication bias was found using Begg’s test for the studies that reported all-cause mortality (P = 0.19) and composite outcomes (P = 0.46), and the Begg’s funnel plots were displayed in Figure S1 and Figure S2, respectively.

### Effect of beta-blockers on hospitalization

**Figure 3:**
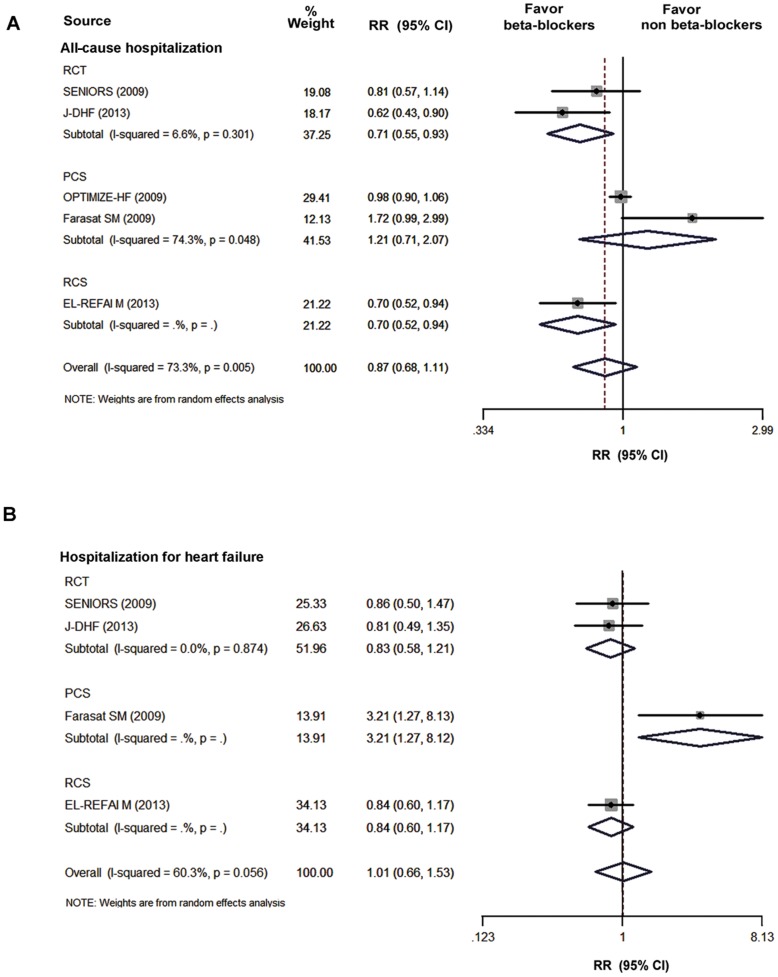
The pooled analysis of hospitalization in beta-blockers group versus non-beta-blockers group. A: All-cause hospitalization; B: HF hospitalization CI, confidence interval; PCS, prospective cohort study; RCS, retrospective cohort study; RCTs, randomized controlled trials; RR, relative risk.

Figure 3 shows the pooled analyses of the included studies that reported the outcomes of hospitalization. Five studies provided the data of all-cause hospitalization. The pooled analysis showed that the beta-blockers treatment did not improve the risk for all-cause hospitalization (I^2^ = 73.3%, RR, 0.87; 95% CI, 0.68 to 1.11; P = 0.26, Figure 3A) in HFpEF. Similarly, the pooled overall RRs of HF hospitalization did not demonstrate a significant benefit of the beta-blocker treatment (I^2^ = 60.3%, RR, 1.01; 95% CI, 0.66 to 1.53; P = 0.97; Figure 3B). No evidence for the publication bias was found using the Begg’s test for studies reporting all-cause hospitalization (P = 0.81) and HF hospitalization (P = 0.09), and the Begg’s funnel plots were displayed in Figure S3 and Figure S4, respectively.

### Sensitivity Analyses

**Figure 4:**
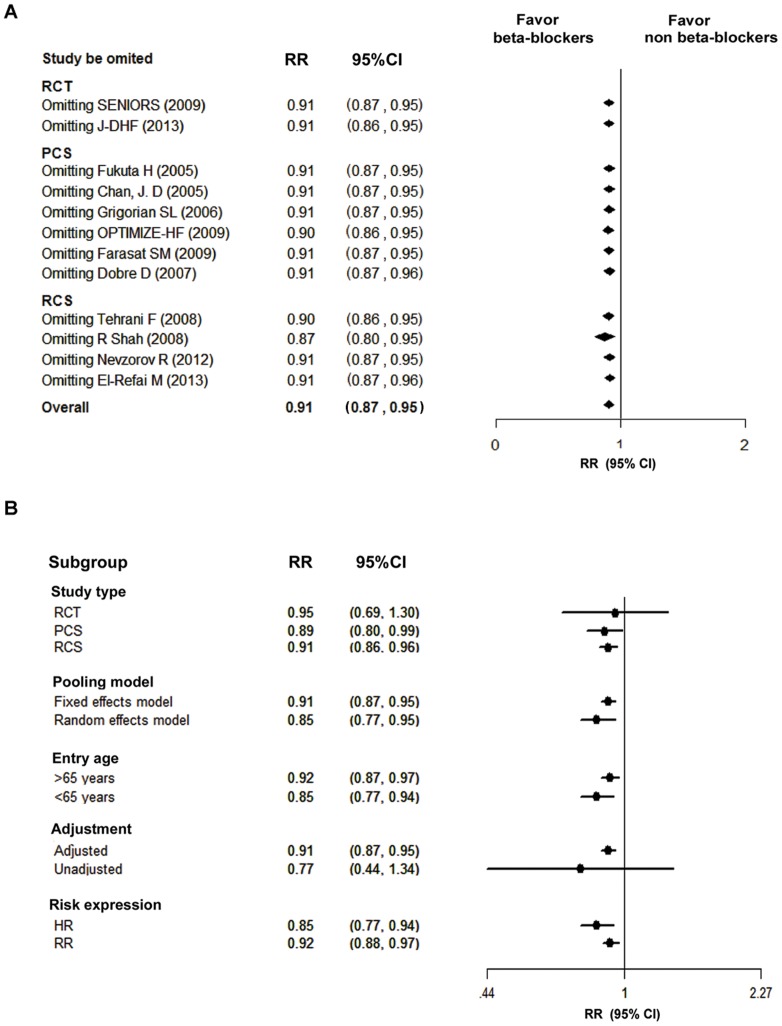
Sensitivity analyses. A: Leave-one-out analysis; B: Subgroup analyses. The adjusted subgroup was those studies that performed multivariate analysis to obtain Relative Risks, and the provided RRs were directly used for pooling analysis. The unadjusted subgroup group was those studies without performing multivariate analysis, and the RRs were calculated by using the primary data. CI, confidence interval; HR, hazard ratio; LVEF, left ventricular ejection fraction; PCS, prospective cohort study; RCS, retrospective cohort study; RCT, randomized controlled trial; RR, relative risk.

We performed leave-one-out sensitivity analysis on all-cause mortality by omitting one study at a time, and found that none of the individual study significantly influenced the pooled estimate of all-cause mortality (Figure 4A). In addition, we conducted subgroup analyses and the results were displayed in Figure 4B. When the pooled analysis of all-cause mortality was performed using random-effect model, a similar result was observed (Figure 4B). A protective effect of beta-blockers was observed when the pooled analysis was limited to those studies that only included elderly patients. However, when the effect estimates were limited to the unadjusted subgroups (those studies performed multivariate analysis to obtain RRs), the pooled analysis did not show a significant effect on all-cause mortality in the patients with HFpEF.

## Discussion

In contrast to the well-reported benefits of the beta-blockers treatment for patients with HFrEF, the effect of beta-blockers exposure in HFpEF remains uncertain. In this meta-analysis involving 21,206 patients, the effect of beta-blockers on the HFpEF with an EF ≥ 40% was firstly evaluated. We found that beta-blocker treatment was associated with a statistically significant reduction in all-cause mortality. However, the prescription of beta-blockers did not significantly improve the hospitalization (all-cause or HF related) or composite outcomes of mortality and hospitalization in HFpEF patients.

It should be emphasized that this meta-analysis was challenged by the differential criteria of the EF cut-off value (range from 35% to 55%) used in the clinical studies investigating HFpEF. In the earlier clinical studies, the cut-off value of EF > 35% was used as the definition of HFpEF [29], [30], while this cut-off value was relatively low and generally not considered “preserved”. In recent years, both the threshold of EF ≥ 50% and EF ≥ 40% were identified as the diagnostic criteria for HFpEF in clinical studies [9], [11]. The heart failure patients with an EF 40% to 50% who had mild systolic dysfunction were characteristically and prognostically similar to those with an EF ≥ 50% [31]. More importantly, recent ACCF/AHA guidelines recognized that the range of 40% to 50% was defined as borderline and intermediate criteria of patients with HFpEF [32]. We, therefore, chose an EF value ≥ 40% as a definition for HFpEF patients in this meta-analysis. To our knowledge, this is the first meta-analysis to evaluate the effect of beta-blockers in HFpEF patients with an EF ≥ 40%.

This meta-analysis reveals that beta-blockers exert a significantly protective effect on all-cause mortality reduction in HFpEF patients. The mortality benefit associated with beta-blockers in this analysis was largely driven by the results of Shah R et al [8]. However, the protective effect still remained after removing this study using the sensitivity analysis. Furthermore, both fixed and random effects models in the pooled analysis shows the significantly similar benefit of the beta-blockers treatment. In addition, this protective effect was noted as well when the pooled analysis was limited to the studies that only included elderly patients. Accordingly, the conclusion that the treatment of beta-blockers reduces all-cause mortality in patients with HFpEF (EF≥40%) is fairly reliable.

The mechanism of the beta-blockers treatment that exerts benefits on all-cause mortality in patients with HFpEF has not been precisely clarified. It might be mainly due to the anti-hypertensive effect, the arrhythmic-risk reduction, and the myocardial perfusion improvement. Previous studies have found that hypertension is the most important cause of HFpEF [33]. Therefore, beta-blockers, as effective anti-hypertension drugs, could exert anti-hypertensive effect and improve the survival of the patients with HFpEF. Additionally, the patients with HFpEF usually have a history of ischemic heart diseases and atrial fibrillation [32]. HFpEF in this condition may benefit from the beta-blockers treatment via controlling ventricular rate, improving myocardial metabolism and ventricular remodeling, and reducing arrhythmic-risk and acute coronary events. The ESC guidelines mentioned that an adequate treatment of hypertension, myocardial ischemia and tachycardia should be recommended to the patients with HFpEF [34].

It is interesting that the protective effect of beta-blockers on HFpEF regarding all-cause mortality is significant, but all-cause (or HF related) hospitalization is not. The lack of the reduction in hospitalization in this meta-analysis is probably due to the following reasons. First, the patients with HFpEF were elderly and typically characterized by multiple non-cardiac or/and cardiac comorbidities. Previous studies demonstrated that the incidence of non-cardiac related hospitalization in HFpEF was much higher, while the incidence of HF hospitalization in HFpEF was lower compared to HFrEF [34]. The comorbidity of diabetes mellitus or COPD might affect the effect of non-selective beta-blockers. These drugs could increase insulin resistance or cause bronchial constrictions. It is possible that the patients with HFpEF hospitalization for diabetes mellitus and/or COPD may not benefit from the beta-blocker treatment. Thus, the higher hospitalization remains. Second, there is very limited data regarding the hospitalization available to produce a meaningful finding. With the clinical studies increase, a significant outcome regarding the hospitalization, in particular HF-related hospitalization, will be reported. In the future, more randomized clinical trials are necessary to explore whether the beta-blockers treatment could improve hospitalization in patients with HFpEF.

Although this meta-analysis demonstrated a significant benefit of beta-blockers on all-cause mortality in HFpEF, the recommendations for this treatment in clinical practice should be cautious. This benefit is mainly derived from the observational studies and only 9% risk reduction was observed. More large-scale RCTs in HFpEF are required to assure the protective effect of beta-blockers. Fortunately, a large-scale clinical trial (β-PRESERVE study) aiming at the role of β-blockers (metoprolol) in HFpEF is on the way now [35].

### Study limitations

There are several limitations in our meta-analysis. First, the publication bias may only occur for published articles in English. Second, the available RCTs were underpowered to provide conclusive findings about the effects of beta-blockers on HFpEF due to small sample sizes. Third, the outcomes regarding exercise tolerance, diastolic function and quality of life were not assessed in this meta-analysis. As we know, it is also important to clarify the effect of beta-blockers on the outcomes of symptoms and functional status. However, the available data is too limited to perform a powerful meta-analysis. Furthermore, the application of meta-analytic methods to the observational studies in this meta-analysis may produce inherent biases, including the observational design that has lost the randomization and made the calculation of a single summary effect estimate potentially misleading. However, the credibility could be greatly improved as we performed and reported this meta-analysis according to the reporting Meta-Analyses of Observational Studies in Epidemiology (MOOSE). Finally, there are only two studies (SENIORS and J-DHF trials) in this meta-analysis that provided the specific doses and types of the beta-blockers administrated We could not assess whether the doses and types of beta-blockers affect the effects of beta-blockers on HFpEF.

## Conclusions

This meta-analysis demonstrated that the beta-blockers treatment reduced all-cause mortality in HFpEF patients with EF ≥ 40%, while it did not affect hospitalization. Beta-blockers may be an efficacious therapeutic option for the patients with HFpEF, and further large scale RCTs are urgently required to assert this issue.

## Supporting Information

Figure S1 Begg’s Funnel Plots with Pseudo 95% Confidence Limits for studies reporting all-cause mortality. RR, relative risk; and SE standard error.
(TIF)

Figure S2 Begg’s Funnel Plots with Pseudo 95% Confidence Limits for studies reporting composite outcome. RR, relative risk; and SE standard error.
(TIF)

Figure S3 Begg’s Funnel Plots with Pseudo 95% Confidence Limits for studies reporting all-cause hospitalization. RR, relative risk; and SE standard error.
(TIF)

Figure S4 Begg’s Funnel Plots with Pseudo 95% Confidence Limits for studies reporting heart failure hospitalization. RR, relative risk; and SE standard error.
(TIF)

Table S1 The Quality of Observational Studies Assessed by Newcastle–Ottawa Scale.
(DOCX)

Checklist S1 PRISMA Checklist.
(DOC)

Material S1 This supplementary material included: 1_Primary data (XLSX); 2_Data for Figure 2A(dta); 3_Data for Figure 2Bb (dta); 4_Data for Figure 3A(dta); 5_Data for Figure 3B(R); 6_Data for Figure 4A(dta); 7_Data for Figure 4B(dta); 8_Command for each figure.
(RAR)
