# Supplementary figures and images for: Effects of Beta-Blockers on Heart Failure with Preserved Ejection Fraction: A Meta-Analysis
Source: PLoS One. 2014 Mar 5;9(3):e90555. doi: 10.1371/journal.pone.0090555 (PMC3944014; doi:10.1371/journal.pone.0090555)

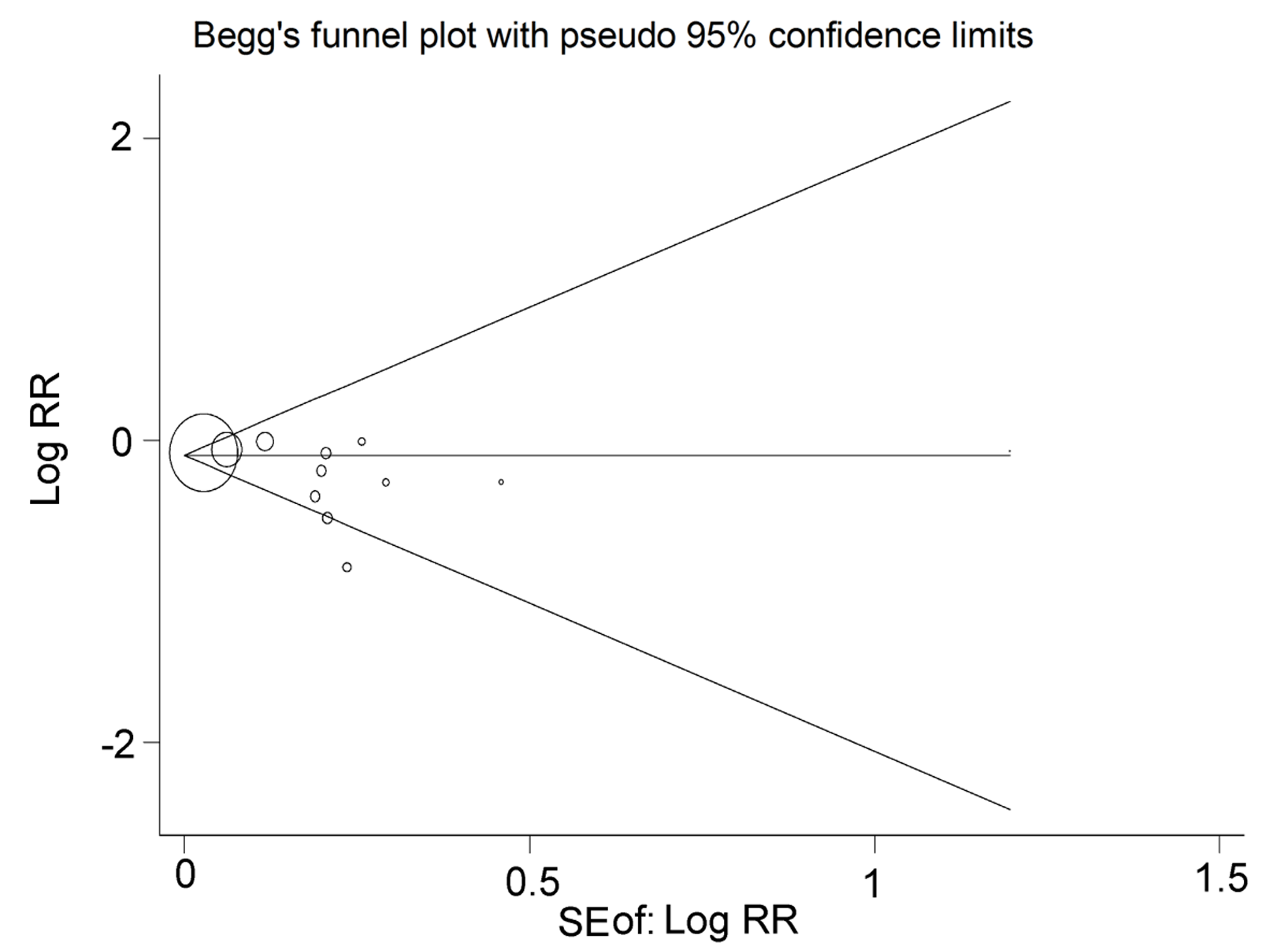

Supplement: Figure S1 — Begg’s Funnel Plots with Pseudo 95% Confidence Limits for studies reporting all-cause mortality. RR, relative risk; and SE standard error. (TIF) [file pone.0090555.s001.tif]

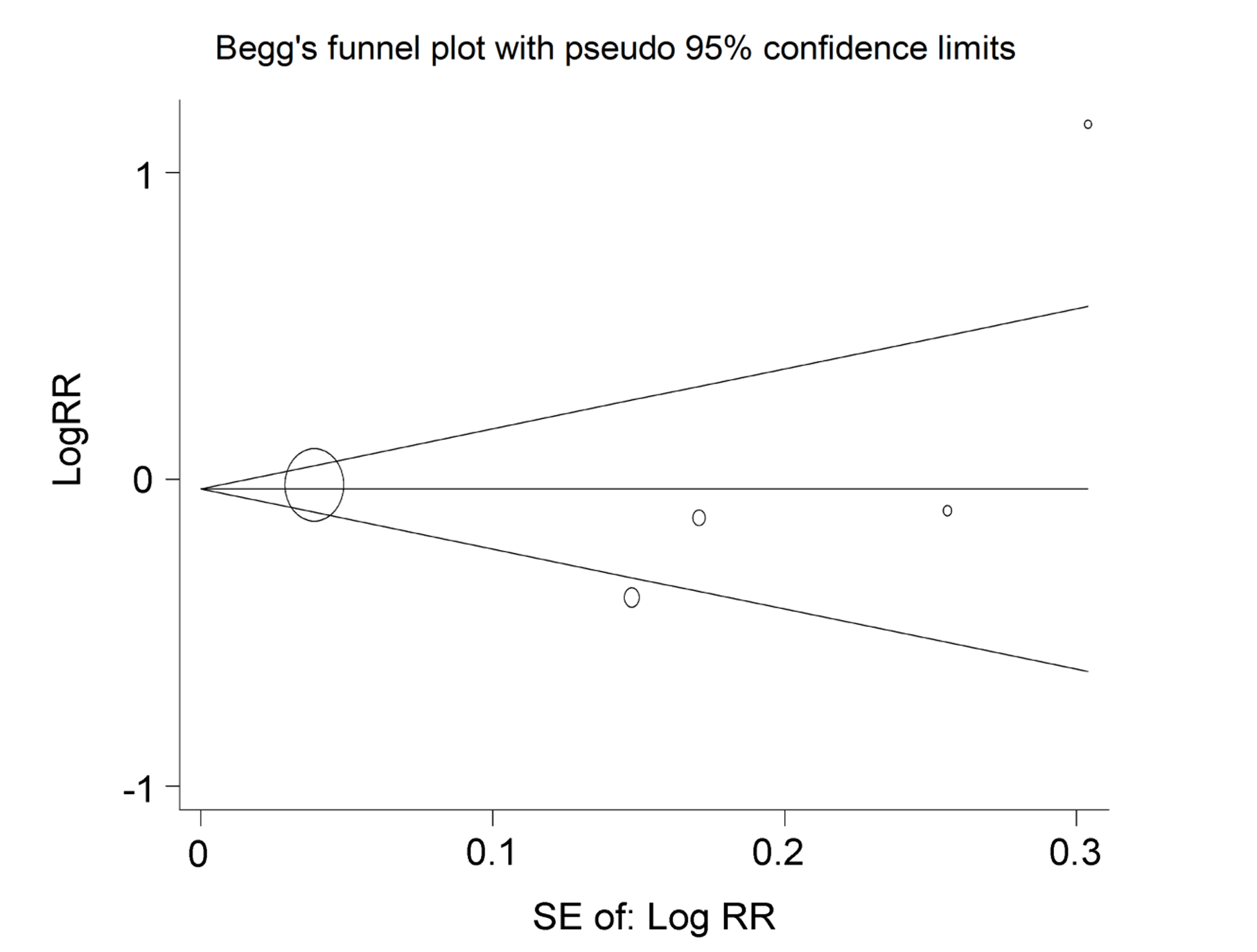

Supplement: Figure S2 — Begg’s Funnel Plots with Pseudo 95% Confidence Limits for studies reporting composite outcome. RR, relative risk; and SE standard error. (TIF) [file pone.0090555.s002.tif]

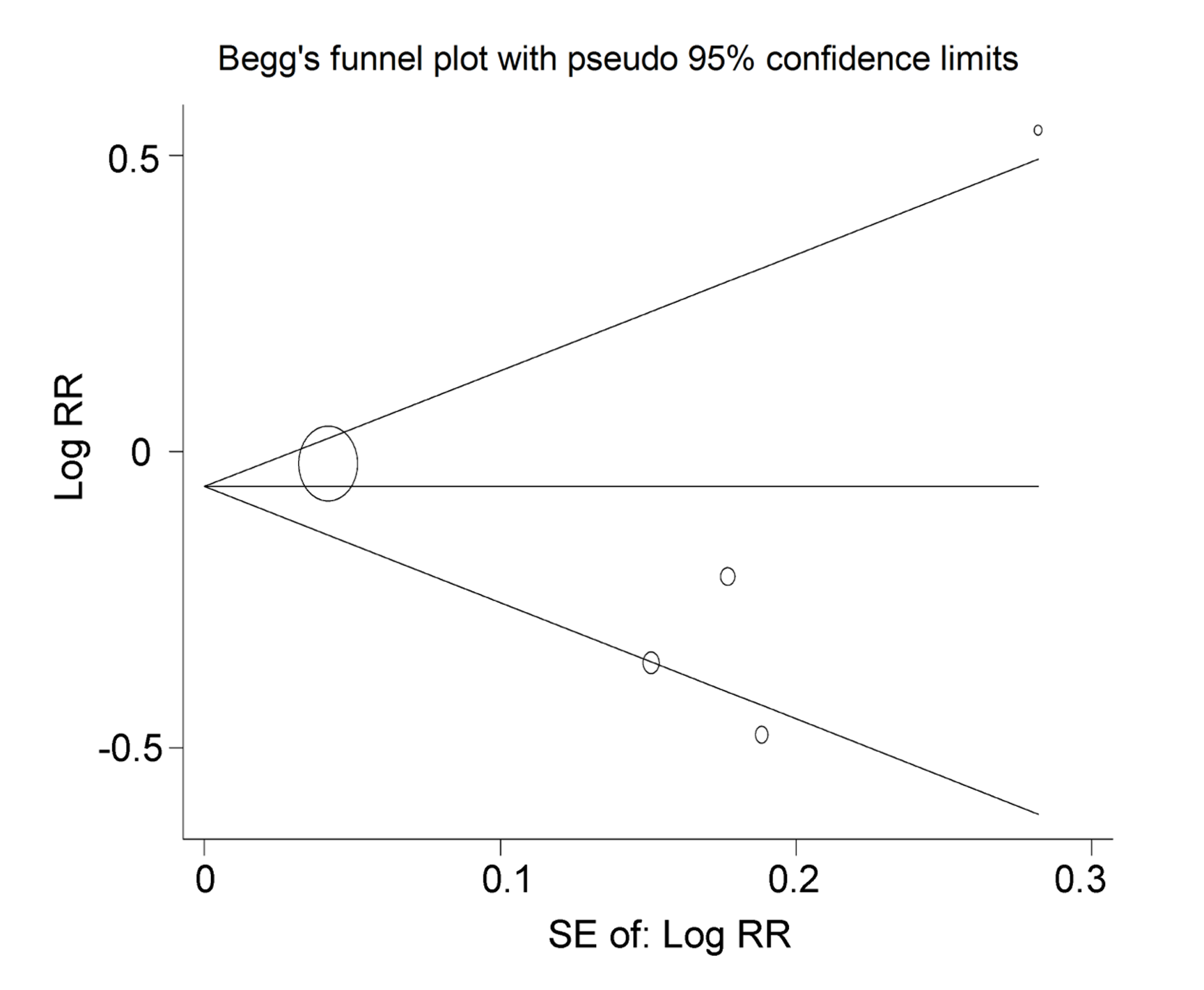

Supplement: Figure S3 — Begg’s Funnel Plots with Pseudo 95% Confidence Limits for studies reporting all-cause hospitalization. RR, relative risk; and SE standard error. (TIF) [file pone.0090555.s003.tif]

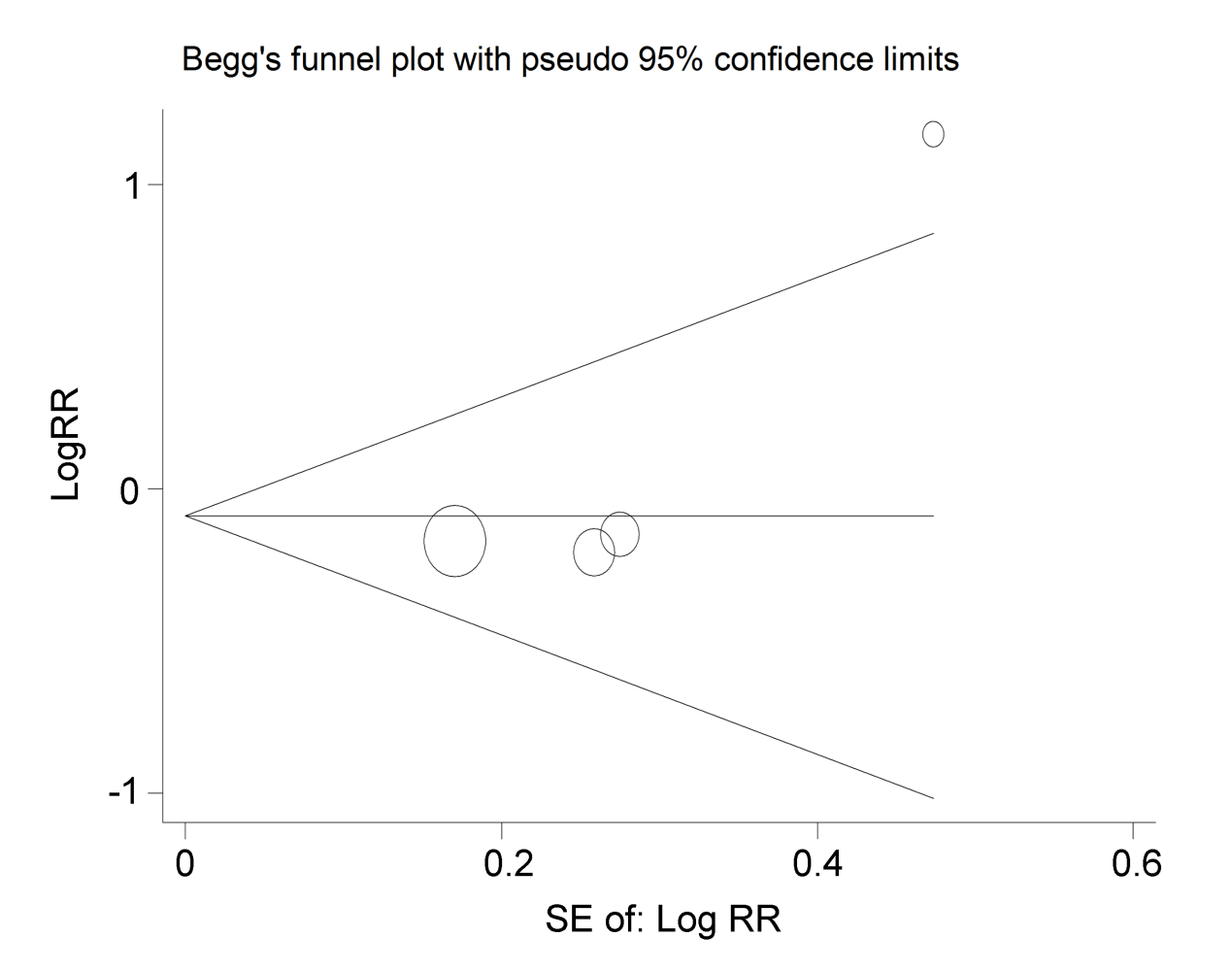

Supplement: Figure S4 — Begg’s Funnel Plots with Pseudo 95% Confidence Limits for studies reporting heart failure hospitalization. RR, relative risk; and SE standard error. (TIF) [file pone.0090555.s004.tif]
