# Supplementary material for: Effects of Beta-Blockers on Heart Failure with Preserved Ejection Fraction: A Meta-Analysis
Source: PLoS One. 2014 Mar 5;9(3):e90555. doi: 10.1371/journal.pone.0090555 (PMC3944014; doi:10.1371/journal.pone.0090555)
Supplement: Table S1 — The Quality of Observational Studies Assessed by Newcastle–Ottawa Scale. (DOCX) [file pone.0090555.s005.docx]

**Table S1: The Quality of Observational Studies Assessed by Newcastle – Ottawa Scale**

|  |  | **Newcastle –Ottawa Scale** | | | | | | | | | |
| --- | --- | --- | --- | --- | --- | --- | --- | --- | --- | --- | --- |
|  |  | **Selection** | | | | **Comparability** | | **Outcome** | | | **Total stars** |
| **Study** | **Year** | **1** | **2** | **3** | **4** | **5A** | **5B** | **6** | **7** | **8** |  |
| **Chan, J. D[24]** | 2005 | B(*) | A(*) | A(*) | A(*) | No | B(*) | B(*) | A(*) | B(*) | 7 |
| **Fukuta H[23]** | 2005 | A(*) | A(*) | A(*) | A(*) | No | B(*) | B(*) | A(*) | B(*) | 7 |
| **Grigorian SL[25]** | 2006 | B(*) | A(*) | C(0) | B(0) | No | No | B(*) | A(*) | B(*) | 5 |
| **OPTIMIZE-HF[26]** | 2009 | A(*) | A(*) | A(*) | A(*) | No | B(*) | B(*) | A(*) | B(*) | 8 |
| **Farasat SM[12]** | 2009 | A(*) | A(*) | A(*) | A(*) | No | B(*) | C(0) | B(0) | B(*) | 6 |
| **Dobre D[7]** | 2010 | B(*) | A(*) | A(*) | A(*) | No | B(*) | B(*) | A(*) | A(*) | 8 |
| **Tehrani F[27]** | 2008 | B(*) | A(*) | A(*) | A(*) | No | No | B(*) | A(*) | A(*) | 7 |
| **R Shah[8]** | 2008 | B(*) | A(*) | C(0) | B(0) | No | No | B(*) | A(*) | B(*) | 5 |
| **Nevzorov R[28]** | 2012 | A(*) | A(*) | A(*) | B(0) | No | B(*) | C(0) | A(*) | A(*) | 6 |
| **El-Refai M[9]** | 2013 | A(*) | A(*) | A(*) | A(*) | No | No | B(*) | A(*) | B(*) | 7 |
